# Robotic-Assisted Dissection of Bulky Lymph Nodes in Cervical Cancer

**DOI:** 10.1155/2014/965698

**Published:** 2014-04-01

**Authors:** Ahmet Göçmen, Fatih Şanlıkan, Muhittin Eftal Avcı

**Affiliations:** ^1^Department of Obstetrics and Gynecology, Ümraniye Education and Research Hospital, Adem Yavuz Caddesi, No. 1 Kadın Hastalıkları ve Doğum Kliniği, 34766 Ümraniye, İstanbul, Turkey; ^2^Departments of Aegean Obstetrics and Gynecology Training and Research Hospital, 35110 Yenişehir, Izmir, Turkey

## Abstract

The resection of bulky lymph node metastases, which may provide a therapeutic benefit, has been proposed in several studies based on laparotomy and laparoscopy. There is no published study in the literature examining the resection of bulky lymph node metastases using a robotic technique. In this report, we presented a patient with cervical cancer who underwent robotic-assisted dissection of bulky lymph nodes. The robotic-assisted operation time was 255 minutes, and the mean console time was 215 minutes. The estimated blood loss was 70 mL. The number of lymph nodes retrieved was 28, and the number of the dissected paraaortic lymph nodes was 13. The number of the lymph node metastases was eight. The bulky lymph nodes which are difficult to be eradicated with standard radiation therapy can be resected with robotic-assisted surgery and successful resection of the lymph nodes can improve the treatment strategy. This minimal invasive technique is safe and feasible for bulky lymph node dissection.

## 1. Introduction

Despite improvements in the screening and treatment modalities for preinvasive cervical lesions, the mortality rate of cervical cancer has not decreased in the last three decades, and cervical cancer has continued to become one of the most common cancers in women, especially those living in developing countries [1]. The most important prognostic factor for patients with cervical cancer is the presence of lymph node metastases [2]. Although importance of this factor has been confirmed in several studies, the International Federation of Obstetrics and Gynecology (FIGO) staging of carcinoma of the cervix depends on clinical findings and does not include lymph node metastasis [3]. The treatment of cervical cancer depends on various factors, such as the FIGO stage of the disease, the histological subtype, the depth of invasion, and the lymph node status [4]. Surgery, radiotherapy, and chemoradiotherapy can be used separately or together according to the situation. Radiotherapy is preferred if lymph node involvement is detected before surgery, but in the case of bulky lymph nodes, there is a conflict regarding the use of radiotherapy. A 50- to 60-Gray dose should not be exceeded because higher doses cause severe toxicity to the neighboring organs, particularly the small bowel. Conversely, this dosage is not sufficient to sterilize bulky lymph nodes >2 cm [5]. The resection of bulky lymph node metastases, which may provide a therapeutic benefit, has been proposed in several studies based on laparotomy and one pilot study performed by laparoscopy [6]. There is no published study in the literature examining the resection of bulky lymph node metastases using a robotic technique. In this study, we report the first case.

## 2. Case

A fifty-four-year-old woman who had been in menopause for four years was admitted to our center with the complaint of postcoital bleeding for one year. Gynecologic examination revealed a 5 cm exophytic fragile lesion arising from the cervix. A biopsy confirmed the diagnosis of at least microinvasive adenocarcinoma. Computed tomography detected bulky lymph nodes at least 3 × 2 cm in size, which were found at the obturator fossa, the common iliac artery bilaterally, and at the right para-aortic region. Radiotherapy was planned after the resection of the bulky lymph nodes. Magnetic resonance imaging before treatment revealed a cervical mass measuring 58 × 31 × 30 mm. There was no invasion through the vagina or the uterus. The tumor extension reached the pelvic sidewall.

**Figure 1:**
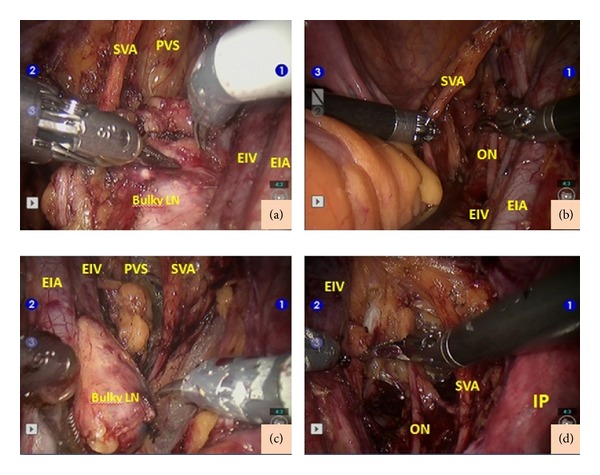
(a) The view of the bulky lymph node on the right obturator fossa. (b) The view after dissection of the bulky lymph node on the right side. (c) The view of the bulky lymph node on the left obturator fossa. (d) The view after dissection of the bulky lymph node on the left side. LN: lymph node; SVA: superior vesical artery; PVS: paravesical space; EIA: external iliac artery; EIV: external iliac vein; ON: obturator nerve; IP: infundibulopelvic ligament.

**Figure 2:**
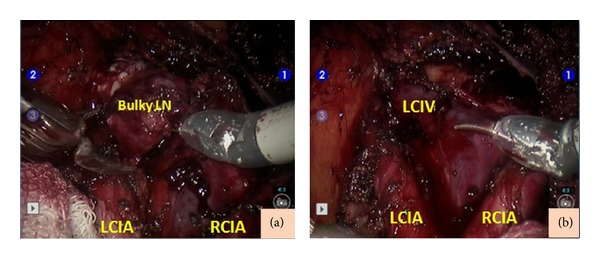
(a) The view of the bulky lymph node on the medial side of the left common iliac artery. (b) The view after the bulky lymph node dissection. LCIA: left common iliac artery; RCIA: right common iliac artery.

Written informed consent was obtained from the patient for the robotic surgery. Mechanical bowel preparation was performed 1 day before surgery. A prophylactic antibiotic and low molecular weight heparin were administered 1 h before the operation. The patient was placed in a low dorsal and steep Trendelenburg position. An appropriate shoulder support was placed to prevent the patient from slipping off the table. The patient underwent insertion of an indwelling Foley catheter. A five-trocar transperitoneal approach was used. The first skin incision for the 12 mm trocar was made 3 to 4 cm above the umbilicus. A direct trocar insertion was performed through the incision, and CO_2_ insufflation was continued until the intra-abdominal pressure was 16 mmHg. All subsequent ports were placed under direct visualization. The 8 mm trocar was introduced into the left upper quadrant of the abdomen, 10 cm lateral to and 1 to 2 cm below the camera port. The 8 mm right trocar was placed symmetrical to the left port. The third robotic instrument port was placed 8 cm lateral to the left trocar and 1 to 2 cm below the left port. A 10 mm assistant port was placed into the left upper quadrant between the camera port and left 8 mm robotic port. V-care (Conmed, USA) was used for uterus manipulation. The bowels were folded into the right paracolic region using a grasper to expose the pelvic anatomy. After the docking procedure, that is, fastening of the patient side cart to the trocars, the camera and endowrist instruments were introduced through the trocars. Monopolar scissors on the right side and bipolar forceps on the left side with either a fenestrated grasper or a Maryland dissector for retraction were used. After the setup, the first author continued the surgery at the console. During the inspection, there was no presence of disease within the abdominal cavity. For the pelvic lymphadenectomy, the paravesical and pararectal regions were identified. After the dissection of the internal and external iliac artery bifurcation, the ureter was retracted medially. There was a bulky lymph node that was 3 × 3 cm in size between the external iliac vein and the superior vesical artery (Figure 1(a)). The psoas muscle was identified and the lymphatic tissue between the external iliac artery and the vein was dissected off the pelvic sidewall. The bulky lymph node was extended to the obturator fossa. The obturator space was entered by reflecting the external iliac vein and the bulky lymph node medially, and the bulky lymph node was dissected from the posterior attachment to the external iliac vein and lateral attachment to the pelvic sidewall while preserving the obturator nerve (Figure 1(b)). For the right para-aortic lymphadenectomy, the right ureter and the psoas muscle were identified after the dissection of the peritoneum over the right common iliac artery. A fenestrated grasper in the fourth arm was used to retract the ureter out of the operative field. There was a bulky lymph node on the medial side of the right common iliac artery that was 2 × 3 cm in size. The lymph node was sent for frozen-section examination, and the result revealed the metastasis of the squamous cell carcinoma. The operation was followed by the pelvic and para-aortic lymphadenectomy procedure on the left side. There were two bulky lymph nodes on the left side; one was on the medial side of the left common iliac artery (Figure 2(a)) and the other was in the left obturator fossa (Figure 1(c)). The sizes of the bulky lymph nodes were 3 × 2 cm and 2 × 2 cm, respectively. The bulky lymph nodes were removed with no complication (Figures 1(d) and 2(b)). The dissected lymph nodes within the endobags were removed through the assistant port.

The robotic-assisted operation time was 255 minutes, and the mean console time was 215 minutes. The estimated blood loss was 70 mL. The hospital stay of the patient was three days. The number of lymph nodes retrieved was 28, and the number of the dissected para-aortic lymph nodes was 13. The number of the lymph node metastases was eight. The operation was completed with no conversion to laparotomy. Radiochemotherapy was planned after the surgical intervention.

## 3. Discussion

In spite of other common gynecologic malignancies, cervical cancer is staged clinically and the actual FIGO clinical staging system is usually considered inadequate due to ignorance of the lymph node status. In approximately 30% of the patients with locally advanced cervical cancer, variations occur between the FIGO clinical staging system and surgical/histopathological findings [7]. To address this inadequacy and to allow individualization of therapy, pretreatment surgical staging carried out by either laparotomy or laparoscopy has been used. Nevertheless, there is some debate about the routine use of pretreatment staging lymphadenectomy, including the necessary delay in the inception of primary radiochemotherapy and the increased risk of operative morbidity. The laparoscopic approach decreases the morbidity rates, but it does not eradicate the cancer [8]. However, recent studies in the literature indicate that pretreatment surgical staging has resulted in modification of the therapy in 18–44% of this group of patients, without significant surgical morbidity, resulting in postponing consecutive radiochemotherapy. Moreover, some researchers have affirmed the therapeutic effect and survival benefit associated with the complete resection of positive lymph nodes, especially bulky nodes that would otherwise be difficult to eradicate with standard radiation therapy [9]. Dose escalation to 75 Gray is necessary to sterilize a large tumor volume (>2 cm); however, this dose is intolerable for the adjacent organs, especially the small bowel [5]. The least standardized treatment procedure in gynecological oncology is surgery due to various factors, such as the anatomy of the patient, differences in the surgical modality, difficulty in measuring the extent of the operation, and inadequate descriptions of some of the surgical procedures and technologies used [10]. The removal of bulky lymph nodes by laparotomy was described by Tozzi et al., who reported the laparoscopic debulking of bulky lymph nodes in women with cervical cancer [6]. The use of laparoscopy is widely accepted in gynecological oncology. Robotic-assisted laparoscopy provides us with a new minimally invasive surgical procedure to overcome the difficulties of conventional laparoscopy and introduce several advantages, including superior visualization, mechanical improvements, stabilization of the instruments within the surgical field, and improved ergonomics for the surgeon.

In summary, we could not identify any case report in the literature regarding bulky lymph node dissection using a robotic technique. The bulky lymph nodes which are difficult to be eradicated with standard radiation therapy can be resected with robotic-assisted surgery and successful resection of the lymph nodes can improve the treatment strategy. This minimally invasive technique is safe and feasible for bulky lymph node dissection.
